# Accumulation of Trace Metal Elements (Cu, Zn, Cd, and Pb) in Surface Sediment via Decomposed Seagrass Leaves: A Mesocosm Experiment Using *Zostera marina* L.

**DOI:** 10.1371/journal.pone.0157983

**Published:** 2016-06-23

**Authors:** Shinya Hosokawa, Susumu Konuma, Yoshiyuki Nakamura

**Affiliations:** 1 Marine Environmental Information Group, Port and Airport Research Institute, 3-1-1 Nagase, Yokosuka, Kanagawa 239–0826, Japan; 2 Coastal and Estuarine Environment Research Group, Port and Airport Research Institute, 3-1-1 Nagase, Yokosuka, Kanagawa 239–0826, Japan; Sun Yat-Sen University, CHINA

## Abstract

Accumulation of Cu, Zn, Cd, and Pb in the sediment of seagrass ecosystems was examined using mesocosm experiments containing *Zostera marina* (eelgrass) and reference pools. Lead was approximately 20-fold higher in the surface sediment in the eelgrass pool than in eelgrass leaves and epiphytes on the eelgrass leaves, whereas zinc and cadmium were significantly lower in the surface sediment than in the leaves, with intermediate concentrations in epiphytes. Copper concentrations were similar in both the surface sediment and leaves but significantly lower in epiphytes. Carbon and nitrogen contents increased significantly with increasing δ^13^C in surface sediments of both the eelgrass and reference pools. Copper, Zn, Cd, and Pb also increased significantly with increasing δ^13^C in the surface sediment in the eelgrass pool but not in the reference pool. By decomposition of eelgrass leaves with epiphytes, which was examined in the eelgrass pool, copper and lead concentrations increased more than 2-fold and approximately a 10-fold, whereas zinc and cadmium concentrations decreased. The high copper and lead concentrations in the surface sediment result from accumulation in decomposed, shed leaves, whereas zinc and cadmium remobilized from decomposed shed leaves but may remain at higher concentrations in the leaves than in the original sediments. The results of our mesocosm study demonstrate that whether the accumulation or remobilization of trace metals during the decomposition of seagrass leaves is trace metal dependent, and that the decomposed seagrass leaves can cause copper and lead accumulation in sediments in seagrass ecosystems.

## Introduction

Seagrass beds, which occur with dense stands of vegetation in coastal and estuarine areas, can be contaminated by trace metals as a consequence of human activity [1] and can be a long-term biogeochemical sink of trace metals [2]. Seagrasses tend to be tolerant of the trace metal impacts in populations in contaminated areas [3]. However, because trace metals may affect benthic community health (e.g. Simpson et al. [4]) and faunal community structures in the ecosystems [5] because of their toxicity, trace metals in seagrass beds would be harmful pollutants in such ecosystems.

Plant litter and related biofilms have been suspected of having an important role in the cycling and biotransfer of trace metals in freshwater ecosystems [6]. In seagrass ecosystems seagrass leaves and epiphytes, which attach to the surface of seagrass leaves, are a major productive part of plants [7, 8], and the leaves can be stored efficiently in dense seagrass beds [9–11]. Because they can contain high concentrations of trace metals, at concentrations correlating to environmental concentrations [12, 13], they are suggested to have significant roles in the cycling of trace metals [14–16]. In addition, accumulation and remobilization of trace metals that occur in the leaves after shedding would be a key process of their biogeochemical deposition in natural seagrass beds. However, disturbances by waves and currents make it difficult to clarify their mechanisms in the natural field.

Few previous studies have focused on estimations of the variation in trace metal concentrations in seagrass beds. Lyngby and Brix [17] examined leaf decomposition of the seagrass *Zostera marina* (eelgrass) in a flow-through system to test the effects of shed leaves on the beds. Their results explain that the sedimentation of dead eelgrass leaves had higher concentration of lead and chromium and lower concentrations of cadmium than green, living leaves in natural eelgrass beds in the Limfjord, Denmark. However, their results could not explain the behaviour of zinc. The insufficient explanation for zinc may be the result of their experiments being performed using experimental seawater, which may have differed from that in the field, and by the complex conditions in the field that may not have been simulated in the experimental system.

Mesocosm experiments simulate natural field conditions. The mesocosm experiment using pools protected from physical disturbances have the advantage that we can extract biological, chemical, and biochemical interactions in individuals, populations, communities, and ecosystems. For seagrasses, mesocosm experiments have clarified the effects of water temperature on the life span of leaves in a seagrass population [18], species interactions in the community associating with seagrass [19], and the effects of nutrients in the water column and sediments on seagrass growth in seagrass ecosystems [20–22]. By using mesocosm experiments, the cycling mechanisms of trace metals in seagrass beds via the leaves may be revealed.

In this study, we focused on the sinking of Cu, Zn, Cd, and Pb in seagrass bed, because their fate and toxicity is commonly focused in seagrass beds [1]. We hypothesized that these trace metal could accumulate in the sediment in seagrass beds because of the effects of trace metals contained in the leaves before shedding and because of the accumulation and remobilization processes after the leaf shedding. To test these hypotheses, we performed two independent mesocosm experiments; one using a seagrass bed and the other using a bed without seagrass. We tested our hypotheses by comparing concentrations of these trace metals in the sediments in these mesocosm pools and by examining the accumulation and remobilization processes of the trace metals in the decomposed leaves in the mesocosm pool with seagrass. The seagrass *Zostera marina* (eelgrass), which is a common species in the northern hemisphere [23] and commonly used for research on trace metal dynamics [1], was used for these tests as the model species of seagrass.

## Materials and Methods

### Mesocosm experiments

#### Design of mesocosm facilities

**Fig 1:**
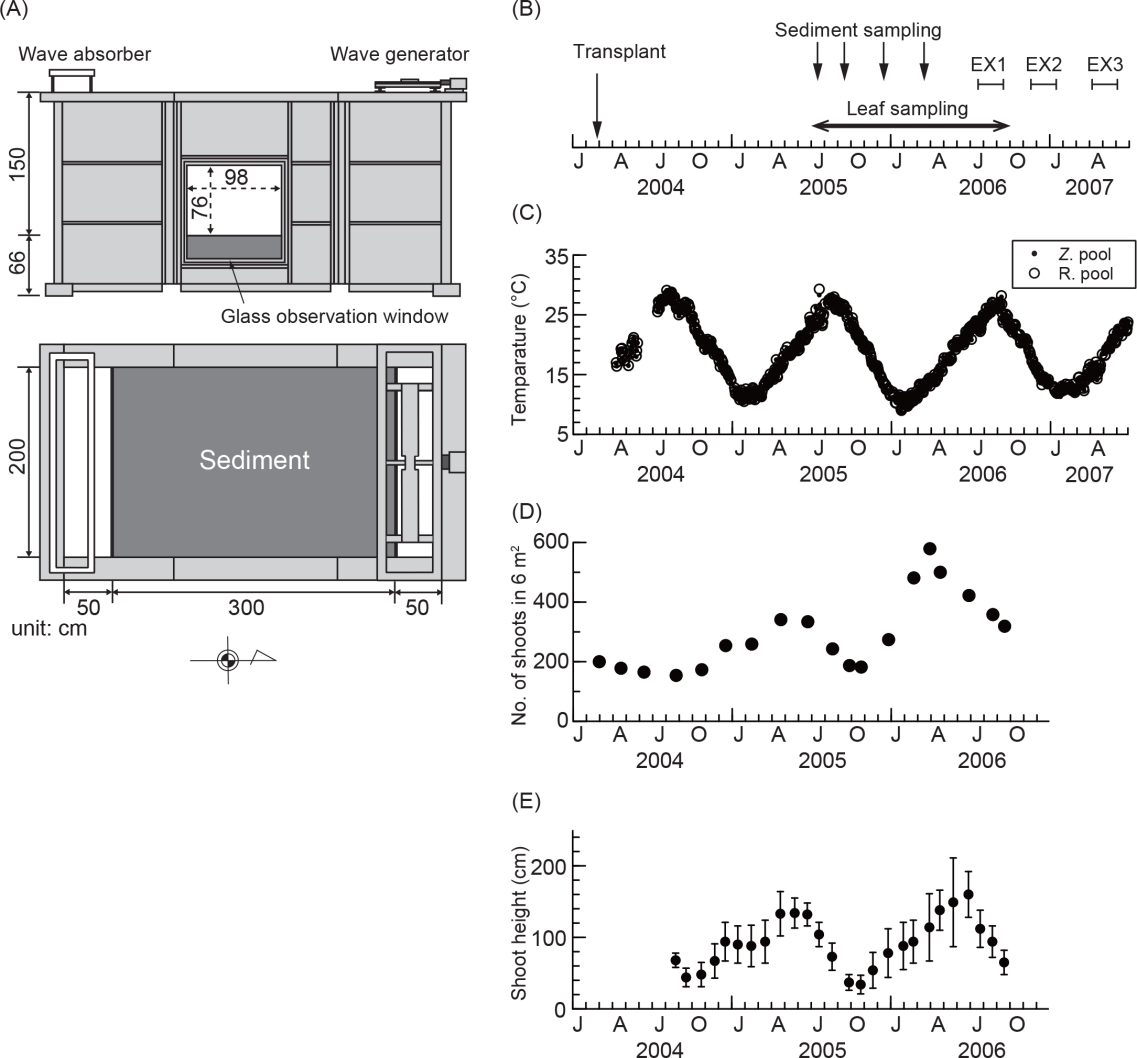
Summary of mesocosm experiments. (A) Side and plane views of the eelgrass pool, (B) time series of experimental schedules, (C) water temperature in the eelgrass pool (Z. pool) and reference pool (R. pool), (D) total number of eelgrass shoots in the eelgrass pool, and (E) the shoot height of eelgrass. Closed circles and error bars for the shoot height indicate the mean and 1 standard deviation (n = 18 per month), respectively.

Mesocosm experiments were conducted using two independent systems at the Port and Airport Research Institute located on Kurihama Bay (35°13ʹN, 139°43ʹE), where a natural eelgrass bed located [24], at the mouth of Tokyo Bay, Japan. Each system had an indoor experimental pool made of fiber-reinforced plastic, which was 3 m long and 2 m wide, and with 1.5 m depth above sediment (Fig 1A). Sandy sediment, with 0.13 mm median particle diameter, was collected from the middle of Tokyo Bay; elements targeted in this study have relatively high concentrations in Tokyo Bay compared to those in sediments in coastal and oceanic waters of Japan [25]. The sediment was placed to a depth of 0.4 m at the bottom of the pool, after homogenizing sediment quality. These pools were housed in a shed with a glass roof, a glass wall facing south, and walls on other sides to avoid atmospheric disturbances (S1 File). The pools used had similar designs, except that one had a window on the east while the other had a window on the west side of the pool. Sunlight came into each pool through the glass roof and wall facing south, and from the side window on each pool.

Eelgrass occurs in coastal areas in Japan [26]. Two hundred eelgrass shoots with rhizomes were collected in February 2004 from the Hashirimizu coast, 4 km northeast of Kurihama Bay, and were transplanted on same day in the pool with a window on the east side. The other pool, with a window on the west side, served as the reference pool and no eelgrass shoots were transplanted.

Seawater without any treatment was obtained for the two experimental systems via a facility pumping from Kurihama Bay and was transported twice each day with every semi-diurnal tide with a 24% exchange rate at 0.5–1.0 m in water depth. Waves were generated with a 5–7 cm height and a 2.0 s period for each pool. The water column temperature in Kurihama Bay ranges from 11°C (February) to 26°C (September) seasonally and is usually over 30 PSU in salinity [27], and does not show distinct seasonal variations in nutrients, which range between 7–23 μM for ammonium, 19–29 μM for nitrate + nitrite, and 0.03–1.65 μM for phosphate (T. Hibino pers. comm.). The salinity and pH of the seawater in mesocosm pools were 29–35 PSU and 7.8–8.3 during this experiment, respectively [18].

#### Monitoring of water temperature and eelgrass growth

Samplings and experiments started 1.5 years after transplantation to permit the plants to recover from the stress of transplantation (Fig 1B and S1 File). Water temperature was monitored, after the transplantation in the eelgrass and reference pools, using a water quality meter (U-21XD, Horiba, Japan). The total number of eelgrass shoots and shoot height in the eelgrass pool were measured to assess eelgrass growth after the transplantation by the end of the samplings. The total number of shoots was counted every one or two months. The height of eelgrass shoots was measured every month for 18 randomly chosen shoots in the pool. Emerged macroalgae were removed each day eelgrass growth was monitored. Eelgrass was not present in the reference pool during this study.

#### Sediment sampling

Sediment was sampled in July, September, and December 2005, and March 2006 (Fig 1B) from both pools after seawater levels decreasing in calm conditions to approximately a 10 cm depth above the sediment. Sediment was sampled using a cylindrical core, 2.9 cm in diameter and approximately 10 cm in length. Surface sediments of 1 cm thickness were sliced after carefully pushing out the sediment at the top of the core. Bottom sediment between 5 and 7 cm from the top of the core was taken from the sediment using the same technique. If the sample included noticeable organisms, such as eelgrass rhizomes, they were removed from the sample. Three replicate cores of sediment were randomly taken from each pool. However, a sample of bottom sediment in the reference pool in March 2006 was not obtained because of collapse during sampling. The samples were freeze-dried after sampling.

#### Eelgrass leaf and epiphytes sampling

Eelgrass leaves and epiphytes were sampled from the eelgrass pool every month between July 2005 and September 2006 (Fig 1B). Five replicate shoots were sampled randomly. Because it was necessary to standardize leaf age so as to avoid variations in trace metal elements due to age differences [14, 17], we defined the third-youngest leaves as the standard age because they were less damaged and had less epiphyte growth than more mature leaves [18], and obtained the age leaf from the shoots. However, leaves were not obtained in September 2005 because there was no plant having the third-youngest leaves because of short leaf life span in the month [18].

Samples were freeze-dried immediately after sampling. Epiphytes were obtained from samples of eelgrass leaves taken from October 2005 to September 2006. The epiphytes were gathered after the freeze-drying because of easy removal from the leaf by desiccation. Because the mass of epiphytes obtained from an eelgrass leaf was not sufficient for subsequent analyses, epiphytes obtained from all leaves sampled within a month were pooled for analysis. In total, 10 monthly pooled samples were obtained. Here, we define an eelgrass leaf without epiphyte removal as ‘eelgrass leaf with epiphytes’ and with epiphyte removal as ‘eelgrass leaf’.

#### Decomposition experiment

Three separate experiments of decomposing eelgrass leaves with epiphytes were designed and each was run for approximately eight weeks in the eelgrass pool (S1 Table). For the first experiment (EX1), eelgrass leaves with epiphytes shed on the sediment surface but before fragmented were randomly sampled. However, because the leaves shed on the sediment surface may have started to decompose, the third-youngest leaves with epiphytes were sampled from live shoots for other experiments, EX2 and EX3. Collected leaves were placed into a mesh bag system in each experiment. The mesh bag system had an inside mesh bag, 27 cm × 20 cm in size and a 2-mm mesh size to sieve leaf fragments, and an outside mesh bag, with <1 mm mesh size, to prevent contamination by fragments from outside of the mesh bag system. The mesh bag system was suspended in the water column in the eelgrass pool during each experiment. Five replicates were sampled from the mesh bag on each sampling day (S1 Table). During sampling, a few leaves that maintained their shape in the inside mesh bag were randomly taken. However, if leaves in the bag were broken due to decomposition, their fragments were randomly taken as a sample. These samples were freeze-dried immediately.

Because water temperature is a key factor of the epiphyte growth on eelgrass leaves in the mesocosm pool [18], the temperature that the experimental leaves experienced before they were sampled was calculated. Because the age of leaves for EX2 and EX3 were obviously the third-youngest, the number of days that experienced temperature was calculated to be 37 by multiplying the average number of days in the plastochrone interval in the mesocosm pool (14.9 days [28]) by 2.5, which is the average plastochrone interval for the third-youngest leaf. Although the leaves for EX1 were sampled from the shed leaves on sediment, the age of the leaves was also assumed to be the third-youngest. Thus, its days of experienced temperature were calculated by the same rule as for EX2 and EX3.

### Chemical analysis

Samples of eelgrass leaves, epiphytes, decomposed leaves, and sediment were analyzed for Cu, Zn, Cd, and Pb after homogenization. Approximately 200 mg of epiphytes and decomposed leaf samples were taken for analysis and digested at 600 W for 30 min in 2.5 mL of high purity nitric acid (Kanto Chemical Supplies, Japan) using a microwave (MDS-2000, CEM, Matthews, North Carolina, USA). For eelgrass leaves, approximately 50 mg samples were digested at 140°C for 100 min in 3 mL of high purity nitric acid (Wako Supplies, Japan) using a microwave digestion system (Speedwave 2, Berghof, Eningen, Germany). Sediment samples of approximately 5 g were digested at 150°C for 5–6 h in a high purity acid (Kanto Chemical Supplies, Japan) mixture containing 30 mL nitric acid, 20 mL hydrochloric acid, and 5 mL perchloric acid.

The target metals Cu, Zn, Cd, and Pb in the digests were analyzed using inductively coupled plasma mass spectrometry for epiphytes and decomposed leaves (HP-4500, Agilent Technologies, Santa Clara, California) and for eelgrass leaves (Agilent 7500C, Agilent Technologies, Santa Clara, California). The sediment metals were analyzed using inductively coupled plasma atomic emission spectrometry (Z-8200, Hitachi, Tokyo, Japan). The QA/QC was monitored using a series of internal quality control standards.

Blanks were analyzed together with samples. The detection limits of the procedure were calculated from blanks in each sequence. Detection limits were generally sufficiently low that differences between reference and test locations could be detected. For some eelgrass leaf samples, sufficient mass was not available and some 40% of samples had lead concentrations that were below detection limits (S2 Table). Nevertheless, this did not affect the overall data interpretation. Less than 2% of cadmium concentrations in eelgrass leaves were below detection limits. Trace metal concentrations were expressed in micrograms of element per gram of dry bulk sample. Epiphytes, decomposed leaves, and sediments were analyzed in the laboratory of IDEA Consultants, Inc. Eelgrass leaves were analyzed in the laboratory of the Port and Airport Research Institute.

Eelgrass leaves, epiphytes, and sediment were analyzed for C and N content to assess their distributions in the eelgrass and reference pools. In addition, the stable isotope ratio of C (δ^13^C) was measured in eelgrass leaves, epiphytes, the initial leaves for decomposition experiments, and surface sediment to determine the partitioning of eelgrass leaves and epiphytes on the decomposed leaves and on surface sediment. Furthermore, the stable isotope ratio of N (δ^15^N) was measured in eelgrass leaves, epiphytes, and the decomposed leaves. All samples were defatted using a methanol/chloroform (2:1, v/v) solution for accuracy of δ^15^N before analysis. In addition, because δ^13^C could not be accurate if samples include carbonates, sediment samples were acidified to eliminate carbonates using 1 M hydrochloric acid [29]. Samples for eelgrass leaves, epiphytes, and the leaves for the decomposition experiment were not acidified, because carbonates must be measured for determination of the weight ratio of epiphytes on the leaves, which were mainly crustose coralline algae [18]. The samples were analyzed using a Delta Plus Advantage mass spectrometer (Thermo Electron, Bremen, Germany) coupled with an elemental analyzer (Flash EA 1112, Thermo Electron) and a Delta Plus mass spectrometer (Thermo Finnigan, Bremen, Germany) coupled with an elemental analyzer (EA 1110, CE Instruments, Milan, Italy). Stable isotope ratios are expressed in δ notation as the deviation from standards as follows:

$$\delta^{13}C\;\mathrm{or}\;\delta^{15}N=\left[R_{sample}/R_{standard}-1\right]\times 10^{3},$$ (1)

where *R* is ^13^C/^12^C or ^15^N/^14^N. Pee Dee Belemnite and atmospheric nitrogen were used as the isotope standards of carbon and nitrogen, respectively.

Because the amount of an epiphyte sample was slight, it was used only for the analysis of δ^13^C and δ^15^N; i.e., the number of samples of epiphytes was 10 for the analysis of δ^13^C and δ^15^N and 9 for chemical analyses of trace metals and of carbon and nitrogen.

### Data analyses

#### Element concentrations in eelgrass leaves, epiphytes, and sediments

To assess the distributions of C and N content and trace metal elements (Cu, Zn, Cd, and Pb) in the eelgrass pool, their means, standard deviations, and standard errors were calculated. These statistics for eelgrass leaves, which were sampled over 15 months, were calculated for a year between October 2005 and September 2006, because epiphytes were obtained in these months. Concentrations in eelgrass leaves, epiphytes, and the surface sediment were compared using the Steel-Dwass test, which is a method to compute the *P*-value for non-parametric multiple comparison, by the pSDCFlig function [30] in R [31]. The Monte Carlo method with 10,000 iterations was used for the computations.

To assess the effects of above-ground productivity on the sediment variances of annual C and N contents, Cu, Zn, Cd, and Pb, variability in the elements among sediments were analyzed by the Kruskal-Wallis rank-sum test (the kruskal.test function in R [31]). Analyzed sediment groups were surface sediments in the eelgrass and reference pools, and bottom sediment in the eelgrass pool. Bottom sediment may not be affected by above-ground productivity. The bottom sediment in the eelgrass pool was used as a representative bottom sediment for the test. In addition, the all elements in the surface their trends with δ^13^C, which can be an indicator for sources of sediment and marine organisms in each pool [29, 32, 33], were tested in each pool by a linear model using the glm function in R [31]: 3 samples every 4 months, 12 samples in total in each pool.

Response variables for these analyses were log transformed, because there was a wide distribution with no negative values. However, normality was also analyzed in the linear models for C and N content and trace metal elements in the surface sediments to confirm the effects of data transformations in statistical results. Detection limits were used as substitutions of these samples for data analyses.

#### Weight ratio of epiphytes on eelgrass leaf

Because the amount of epiphytes on eelgrass leaves may be a key factor in leaf decomposition, the weight ratio of epiphytes on the leaves (WRE; *r*) was determined. However, because it was impossible to measure the WRE in the decomposition experiment samples, it was inferred from elements in the initial leaves for decomposition experiments (IL), eelgrass leaves, and epiphytes that were obtained from the first day of EX1 and the first day of EX2 and EX3 in the previous year.

The WRE was estimated by the element combination of C and δ^13^C and by the combination of C, δ^13^C, N, and δ^15^N to confirm contributions of N and δ^15^N in the estimation, which may be easily loss in eelgrass leaves and epiphytes. Monthly averaged values and standard deviations for eelgrass leaves were used as a source values. However, because epiphytes were not sampled for replication within one month, annual averaged values and standard deviations were used as another source values.

The probability density of the WRE was determined by a stable isotope analysis, which is a Bayesian method assuming variability of parameters in sources and can estimate the ratio including uncertainties using a model fitting technique based on the Markov chain Monte Carlo (MCMC) method [34]. The WREs were estimated by 200,000 iterations and 50,000 burn-ins. The values by the MCMC were drawn every 15 iterations and extracted into a total of 10,000 iterations. Mean, standard deviation, and 95% of a Bayesian credible interval, which has a similar meaning to a classical confidence interval [35], were calculated from the estimated distribution of the probability density of the WRE.

#### Leaf decomposition experiments

Trace metal elements in decomposed leaves were modelled to vary with variations in C content (e.g. Schaller et al. [36]). The metals were assumed to be generated by the following model:

$$\log_{e}y_{ij}=A_{ij}\cdot \beta_{i}+\varepsilon_{ij},$$ (2)

where *y*_*ij*_ is the concentration of Cu, Zn, Cd, and Pb for the sample *j* (1 ~ *n*_*j*_) in the experiment *i* (1 ~ *n*_*i*_), and **A**_*ij*_ is a vector of explanatory variables, described as [1, *C*_*ij*_], where *C*_*ij*_ is the C content for sample *j* in experiment *i*. ***β***_*i*_ is a vector of parameters for explanatory variables in experiment *i*, described as [*β*_1*i*_, *β*_2*i*_]^T^. *ɛ*_*ij*_ is the error. Here, we used *ɛ*_*ij*_ that has a variance *σ*_*i*_^2^ and an independent and identical normal error distribution. Because error distributions for *ɛ*_*ij*_ may give misleading impressions on positive response variables [37], the response variable is the log-transformed *y*_*ij*_. The detection limit for cadmium was used as substitutions of these samples for this data analysis.

The model for the trends of trace metals in decomposed leaves was also a Bayesian method which deals with parameter distributions as probabilities (S2 File). The results will be combined with results of the WRE, which was determined by the Bayesian stable isotope analysis model [34], to estimate C content in IL. Parameters were sampled from posterior distributions by the MCMC with 3 chains and 50,000 iterations after a burn-in of 5,000 iterations per chain. Probability densities of the parameters were drawn every 15 iterations from the total 150,000 MCMC results and extracted 10,000 iterations in total. Mean and 95% of a Bayesian credible interval of parameters were calculated from the distribution of probability density.

Variability in Cu, Zn, Cd, and Pb during the decomposition process was predicted with the following equation;

$$p\left(log_{e}y_{i}^{\prime }|\log_{e}y_{i}\right)=∭p\left(log_{e}y_{i}^{\prime }|\beta_{i},\sigma_{i}^{2},C\right)p\left(\beta |log_{e}y_{i}\right)p\left(\sigma_{i}^{2}|log_{e}y_{i}\right)p\left(C\right)d\beta d\sigma_{i}^{2}dC,$$ (3)

where *y′*_*i*_ is the predicted concentration of Cu, Zn, Cd, and Pb at a given C content. *p*() is the probability of parameter in the parenthesis. *p*(***β***_*i*_| log_*e*_*y*_*i*_) and *p*(*σ*_*i*_^2^| log_*e*_*y*_*i*_) are posterior density of parameters ***β***_*i*_ and *σ*_*i*_^2^ obtained by Eq (1), respectively. *p*(*C*) is the distribution of C content for the prediction target. Here, prediction targets were set at the C content in IL (*C*(0)) and at the lowest C content in each decomposition experiment. *C*(0) is determined by the definition of the WRE (*r*) by the following equation;

$$C\left(0\right)=\left(1-r\right)C_{\mathrm{eelgrass}}+rC_{\mathrm{epiphytes}},$$ (4)

where *C*_eelgrass_ and *C*_epiphytes_ are C content in the eelgrass leaves and epiphytes, respectively. For the prediction at *C*(0), its probability density was estimated by *p*(*r*), *p*(*C*_eelgrass_), and *p*(*C*_epihytes_). *p*(*r*) is the estimated probability density of the WRE. *p*(*C*_eelgrass_) and *p*(*C*_epihytes_) were assumed to have normal distributions with the mean and standard deviation calculated from samples between October 2005 and September 2006. Predicting *p*(log_*e*_*y′*_*i*_ | log_*e*_*y*_*i*_) was performed by 100,000 iterations. Its mean and 95% of a Bayesian credible interval were calculated from the predicted distribution of probability density.

## Results

### Water temperature and eelgrass growth

Water temperature varied annually between 10°C and 30°C during the experiments in both the eelgrass and reference pools (Fig 1C). The number of eelgrass shoots varied annually without disappearance during samplings and experiments (Fig 1D). The minimum number of shoots was 154 in the first summer (August 2004) and the maximum number was 579 in March 2006. The height of eelgrass shoots also varied annually between 34 ± 13 cm in October 2005 (*n* = 18) and 160 ± 32 cm in June 2006 (*n* = 18) (Fig 1E).

### Element concentrations in eelgrass leaves, epiphytes, and sediments

**Fig 2:**
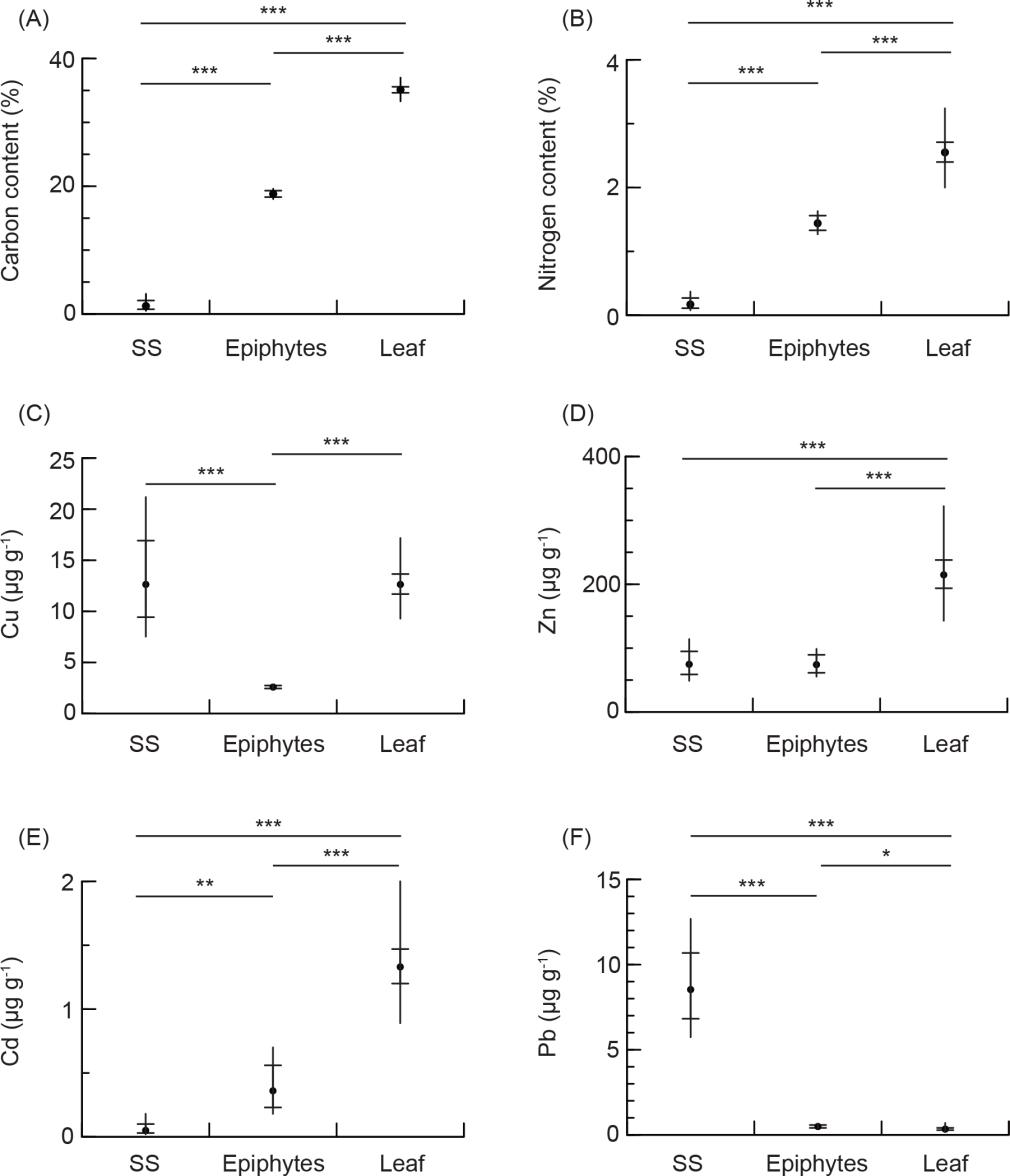
Annual element concentrations in eelgrass leaves (Leaf), epiphytes, surface sediments (SS) in the eelgrass pool. Elemental concentrations are for (A) carbon, (B) nitrogen, (C) copper, (D) zinc, (E) cadmium, and (F) lead. Closed circle and vertical bar are the mean and 1 standard deviation, respectively. The range between horizontal bars on the vertical bar is the 1 standard error. These statistics were calculated from log-transformed data but are shown in normal scale. * *P* < 0.05; ** *P* < 0.01; *** *P* < 0.001.

Concentrations in C, N, Zn, and Cd were significantly greater in eelgrass leaves than in the surface sediment (Fig 2A, 2B, 2D and 2E and S3 Table). Carbon and nitrogen contents and Cd were more than 10-fold greater in eelgrass leaves than in the surface sediment, and was intermediate in epiphytes. Zinc was approximately 3-fold greater in eelgrass leaves than in the surface sediment and epiphytes, which were similar with no significant difference (Fig 2D and S3 Table). Copper concentrations were similar between eelgrass leaves and surface sediment, which were approximately 4-fold greater than in epiphytes (Fig 2C and S3 Table). Lead was similar between eelgrass leaves and epiphytes, and approximately 20-fold lower than in the surface sediment (Fig 2F and S3 Table).

**Fig 3:**
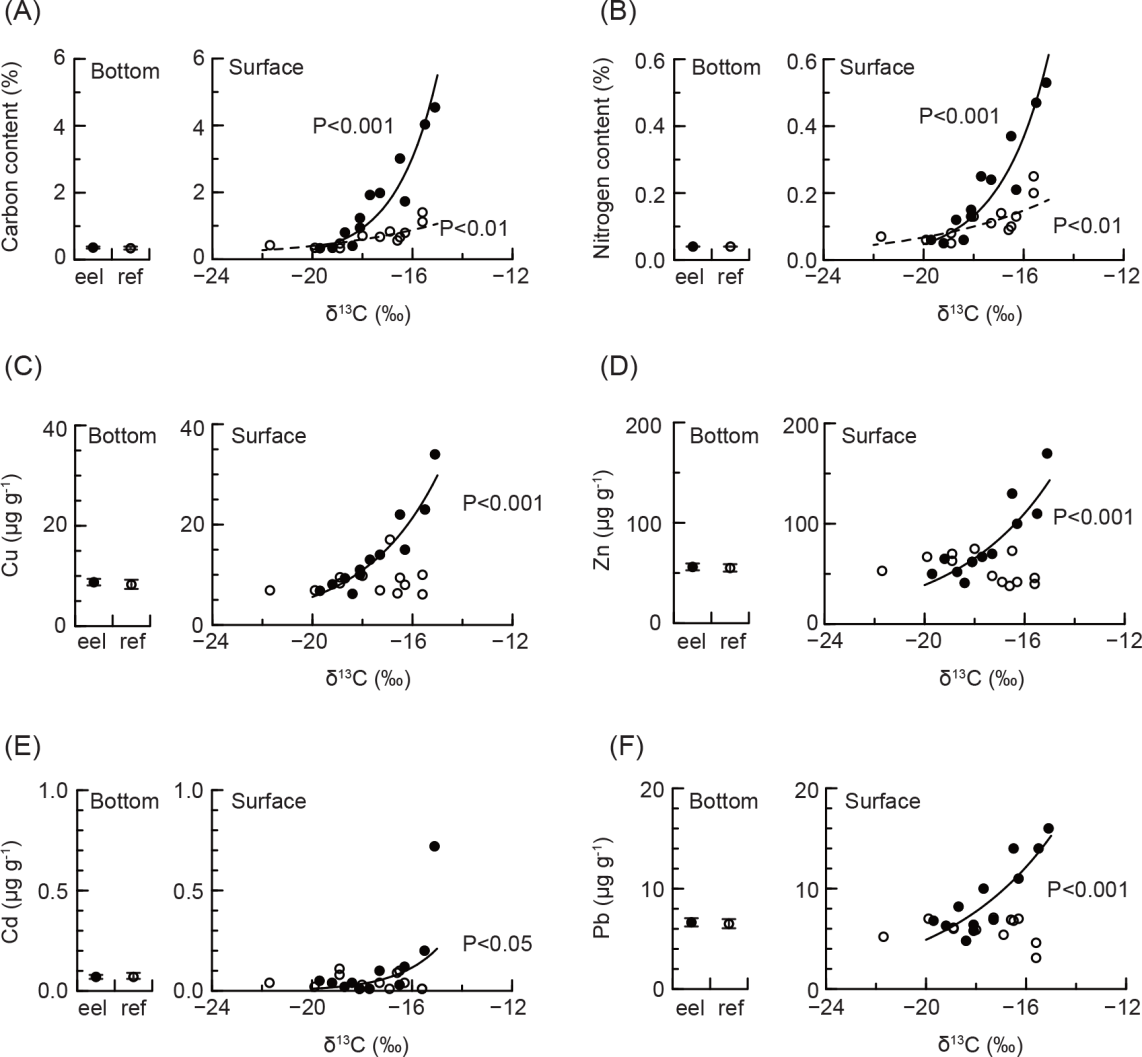
Trend of the element concentrations vs. δ^13^C in the surface sediment and annual concentrations in bottom sediments. Element concentrations are for (A) carbon, (B) nitrogen, (C) copper, (D) zinc, (E) cadmium, and (F) lead. Closed and open circles are the measured data in the eelgrass and reference pools, respectively. Circles and bar in the bottom sediment are the mean and 1 standard deviation in the eelgrass pool (eel) and reference pool (ref). Solid and dashed lines in the surface sediment are the significant regression line for eelgrass and reference pools, respectively. *P*-values are indicated close to the related lines. Analyses were performed for log-transformed data, but results are shown in normal scale.

The carbon stable isotope ratio was −22.2 ± 0.6 (mean ± 1 standard deviation, *n* = 12) in the bottom sediment in the eelgrass pool, −22.1 ± 0.4 (*n* = 11) in the reference pool, −9.0 ± 0.4 (*n* = 4) in eelgrass leaves in September 2005 and −13.4 ± 2.1 (*n* = 5) in July 2006, and −9.7 ± 0.5 (*n* = 10) in an annual average in epiphytes. The carbon stable isotope ratio in the surface sediment ranged between these values (see x axes in Fig 3).

Variations in carbon and nitrogen concentrations were significant among surface and bottom sediments in the eelgrass pool, and surface sediments in the reference pool (Kruskal-Wallis rank-sum test; Kruskal-Wallis *χ*^2^ = 15.2, *df* = 2, *P* < 0.001 for carbon; Kruskal-Wallis *χ*^2^ = 25.3, *df* = 2, *P* < 0.001 for nitrogen). These elements in surface sediment increased significantly with δ^13^C in both pools (Fig 3A and 3B). Their trends were steeper in the eelgrass pool than in the reference pool (S4 Table). Maximum likelihood estimations of C and N in high observed δ^13^C were more than 10-fold in the eelgrass pool than in bottom sediment.

Variations in Cu, Zn, Cd, and Pb concentrations were not significant among surface and bottom sediments in the eelgrass pool, and surface sediments in the reference pool (Kruskal-Wallis rank-sum test; Kruskal-Wallis *χ*^2^ = 5.83, *df* = 2, *P* > 0.05 for copper; Kruskal-Wallis *χ*^2^ = 4.86, *df* = 2, *P* > 0.05 for zinc; Kruskal-Wallis *χ*^2^ = 2.85, *df* = 2, *P* > 0.05 for cadmium; Kruskal-Wallis *χ*^2^ = 5.33, *df* = 2, *P* > 0.05 for lead). However, concentrations in Cu, Zn, Cd, and Pb increased significantly with δ^13^C in the surface sediment in the eelgrass pool, but not in the reference pool (Fig 3C, 3D, 3E and 3F and see S4 Table).

### Leaf decomposition experiments

The temperatures experienced by the leaves in the decomposition experiments were higher in EX1 and EX2 than the leaves in EX3 (S1 Table). The experimental temperature differed from the experienced temperature and was the highest in EX1, lowest in EX2 and moderate in EX3.

**Fig 4:**
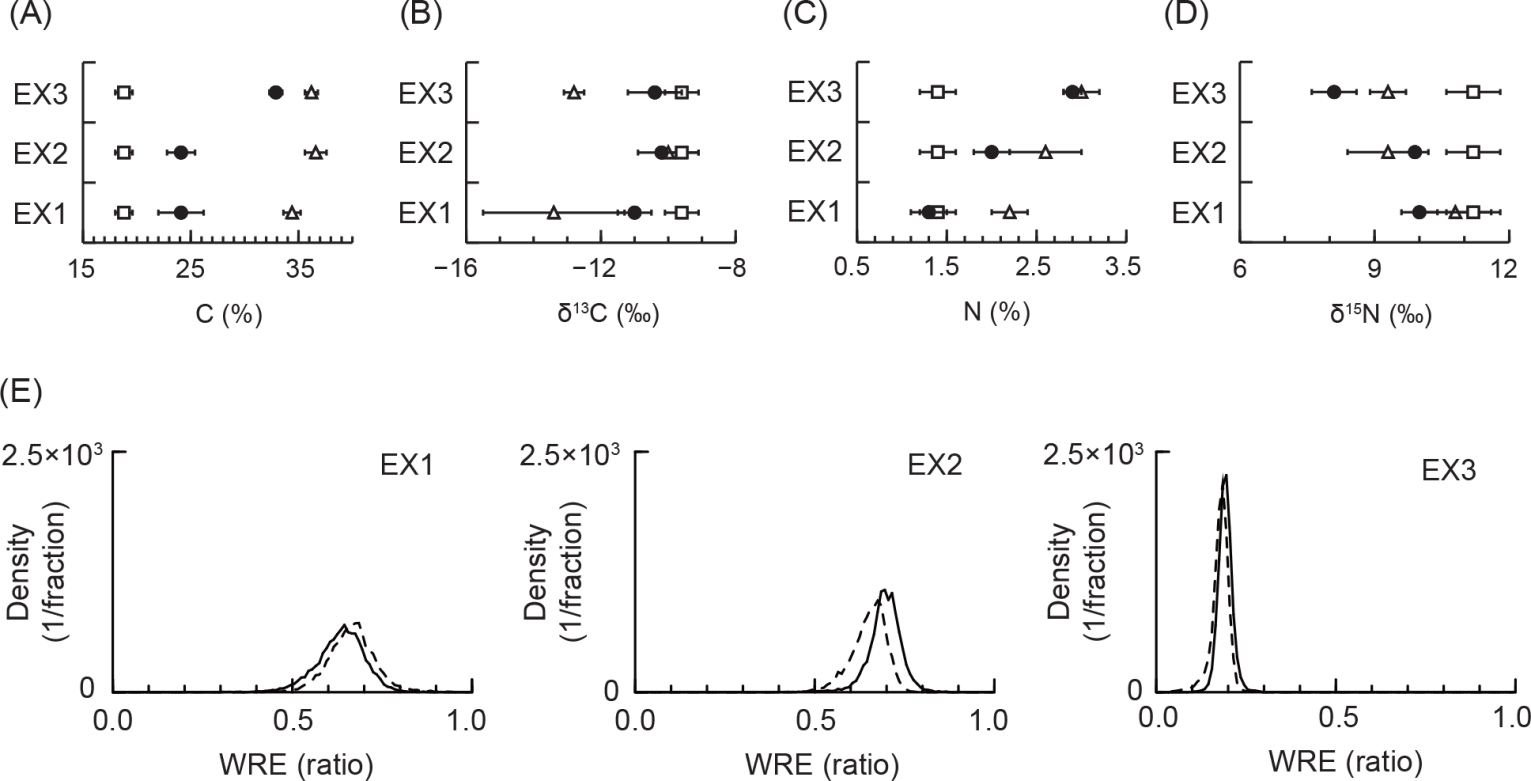
Element concentrations for estimation of weight ratio of epiphytes on eelgrass leaves (WRE) in initial leaves (IL) for decomposition experiments, and the estimation results. (A) C content, (B) δ^13^C, (C) N content, and (D) δ^15^N are the concentrations for WRE estimation in IL. Open triangle and square mean eelgrass leaves and epiphytes, respectively; they are sources for estimating WRE in IL. Closed circle is the IL. Error bar indicates 1 standard deviation. The estimated probability distribution of the WRE is shown in (E). The solid line is the probability density of WRE determined by C content and δ^13^C. The dashed line is the density determined by C, δ^13^C, N, and δ^15^N. The density was shown as 100 fractions between 0.0 and 1.0 for 10,000 iterations.

Carbon content showed approximately 15% differences between eelgrass leaves and epiphytes with relatively narrow standard deviation in each experiment (Fig 4A). Carbon content in IL was within their sources. Differences in distributions of δ^13^C, N, and δ^15^N in eelgrass leaf, epiphytes, and IL were unclear by comparing to those of carbon content (Fig 4B, 4C and 4D).

The WRE estimated by carbon and δ^13^C were 0.634 ± 0.067 (Mean ± 1 standard deviation) with a 0.490–0.754 95% credible interval (C.I.) in EX1, 0.695 ± 0.046 with a 0.597–0.779 95% C. I. in EX2, and 0.192 ± 0.020 with a 0.155–0.232 95% C. I. in EX3 (Fig 4E). The WRE in EX3 was different apparently from those in EX1 and EX2. The WRE estimated by C, δ^13^C, N, and δ^15^N showed similar distributions in the WRE estimated by carbon and δ^13^C.

**Fig 5:**
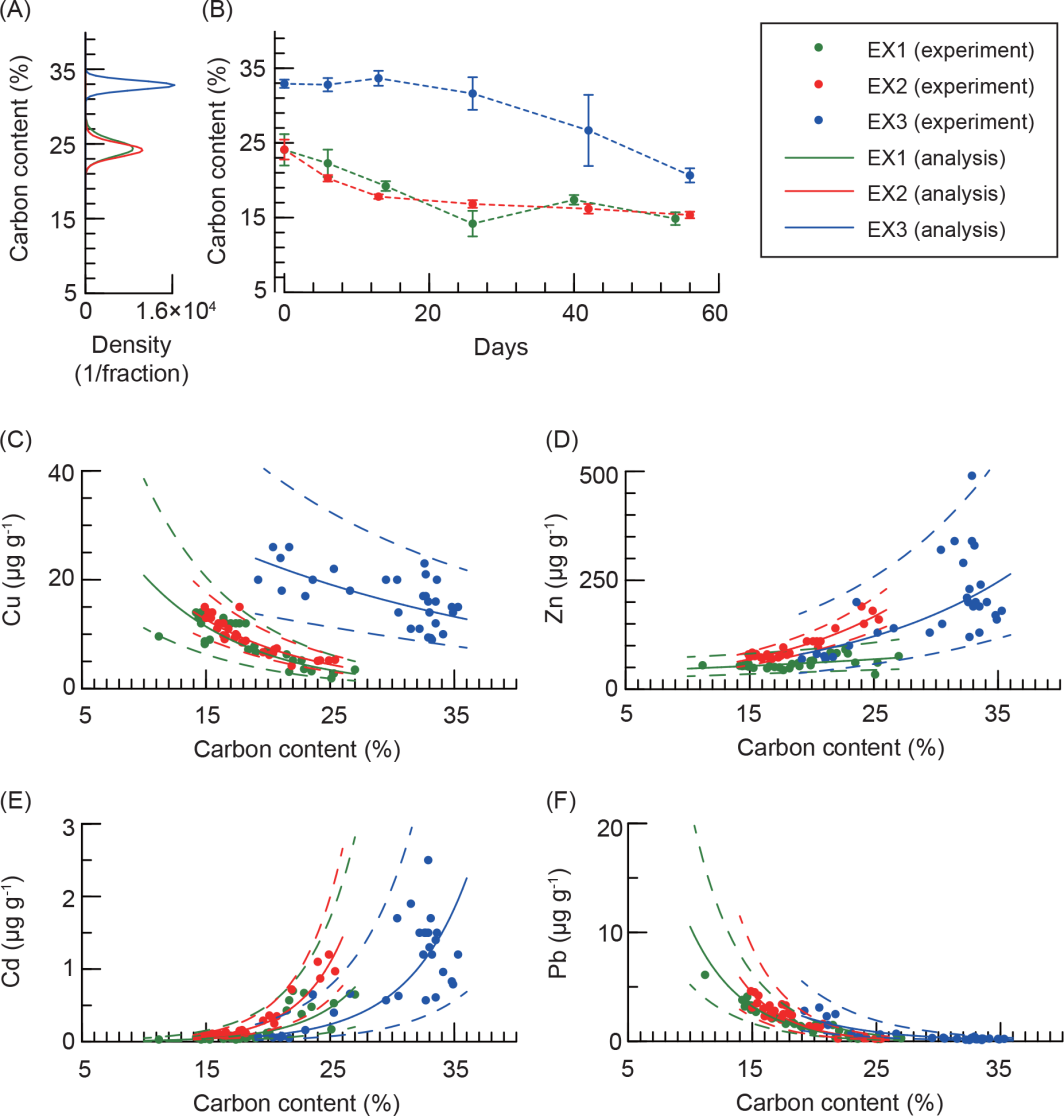
Summary of results for decomposition experiments. (A) Estimated probability distributions of carbon content in initial leaves for decomposition experiments, and (B) experimental results of carbon content vs. experimental days. The density in (A) is shown as 100 fractions between 15 and 40 for 100,000 iterations. The closed circle and error bar in (B) indicate the mean and 1 standard deviation, respectively. Experimental and statistical results for Cu, Zn, Cd, and Pb vs. carbon content are shown in (C), (D), (E), and (F). In these figures, closed circles, and solid and dashed lines indicate experimental results, and the mean and 95% credible interval, respectively. These statistical analyses were performed by log-transformed data, but results are shown in normal scale.

Carbon contents were between 21 and 35% in IL (Fig 5A) and decreased from the IL in each experiment (Fig 5B). The lowest carbon content in leaves during experiments was 11.2% in EX1, 14.9% in EX2, and 19.2% in EX3.

Copper and lead significantly increased with increasing decomposition, i.e. decreasing carbon content (negative *β*_2_, see Table 1), whereas Zn and Cd decreased (positive *β*_2_). For Cu the increase was approximately 2- or 5-fold with decreasing carbon content (Fig 5C, and see S5 Table) and more than 10-fold for lead (Fig 5F and S5 Table), whereas the decrease in concentration of zinc was a few fold (Fig 5D and S5 Table) and more than 10-fold for cadmium (Fig 5E and S5 Table). The decomposition experiment showed an apparent difference in the distribution of copper in EX3 compared to other experiments, whereas differences were not apparent for Zn, Cd, and Pb. This would be caused by the variability in concentrations in eelgrass leaves (zinc and cadmium) and by the relatively extreme variations during decomposition (cadmium and lead).

**Table 1:** Mean and 95% credible interval (CI) of parameters for the model of decomposition experiments.

		*β*_1i_				*β*_2i_				*σ*_*i*_^2^			
		Mean	95% CI			Mean	95% CI			Mean	95% CI		
Cu	EX1 (*i* = 1)	4.23	3.80	—	4.70	−0.120	−0.145	—	−0.097	0.08	0.05	—	0.14
	EX2 (*i* = 2)	4.12	3.79	—	4.45	−0.105	−0.123	—	−0.087	0.03	0.02	—	0.04
	EX3 (*i* = 3)	3.88	3.34	—	4.39	−0.037	−0.054	—	−0.019	0.07	0.04	—	0.12
Zn	EX1 (*i* = 1)	3.59	3.17	—	3.98	0.025	0.005	—	0.047	0.04	0.03	—	0.08
	EX2 (*i* = 2)	2.90	2.65	—	3.15	0.089	0.075	—	0.102	0.01	0.01	—	0.02
	EX3 (*i* = 3)	3.03	2.34	—	3.66	0.071	0.050	—	0.094	0.14	0.08	—	0.24
Cd	EX1 (*i* = 1)	−6.68	−7.55	—	−5.78	0.237	0.189	—	0.283	0.38	0.23	—	0.65
	EX2 (*i* = 2)	−6.81	−7.43	—	−6.18	0.276	0.242	—	0.309	0.08	0.05	—	0.14
	EX3 (*i* = 3)	−6.53	−7.55	—	−5.46	0.204	0.168	—	0.238	0.34	0.20	—	0.57
Pb	EX1 (*i* = 1)	4.73	4.13	—	5.35	−0.238	−0.270	—	−0.206	0.11	0.06	—	0.18
	EX2 (*i* = 2)	5.65	4.80	—	6.41	−0.277	−0.318	—	−0.232	0.11	0.06	—	0.18
	EX3 (*i* = 3)	4.04	3.16	—	4.93	−0.168	−0.198	—	−0.139	0.16	0.09	—	0.27

## Discussion

Our mesocosm experiment showed that concentrations of trace metals increased with an increase in marine organisms in the surface sediment of eelgrass bed. Zinc and cadmium concentrations in eelgrass leaves decreased during decomposition but still remaining higher in concentration than in the original sediments. Conversely, the increase in copper and lead in the surface sediment was caused by the metals in the decomposed eelgrass leaves; more than 2-fold in copper and approximately 10-fold increase in lead was observed in eelgrass leaves during decomposition. These findings show that copper and lead can accumulate in seagrass beds through primary productivity, and the changes in sediment quality may have negative impacts on ecosystems in coastal areas.

### Decomposition processes

The increase in copper and lead in decomposed leaves could be a result of three scenarios: (i) an accumulation in the leaf; and two passive concentration increases, (ii) that sedimentation derived from the water column accumulated in the decomposed leaves and increased the concentration, or (iii) that the increase was a result of tissue loss that did not bind the metals. However, the probability of increasing concentration by sedimentation from water column was low in this experiment because of no significant trend with marine organisms in the surface sediment in the reference pool. In addition, the scenario of tissue loss that did not bind the metals would not be acceptable because a 2-fold increase in sediment metal concentrations requires massive losses of tissues that do not bind to metals. Thus, it is most likely that the concentrations increased by the accumulation of metals in decomposed leaf tissue. Decreases in zinc and cadmium during leaf decomposition could be caused by metals leaching from the leaf by physical and biophysical decomposition.

Accumulation of copper and lead in decomposing leaves may occur through biophysical fragmentations in the leaves, such as shredding by invertebrates, which increases the surface area of the leaves [36], and biofilm development results in a high concentration of metals [6]. These metals detected in seawater (S3 File) may support the idea that the metals accumulated in decomposed leaves originated from the water column. Conversely, the decreases in zinc and cadmium in this experiment suggest that the leaching from decomposing leaves was stronger than their accumulation potentials, in spite of the fact that these metals accumulate in leaves under freshwater conditions [36]. In addition, zinc and cadmium are known to be at relatively low concentrations in dissolved organic matter containing humic substances in comparison to copper and lead [38, 39]. The properties of humic substances may cause different trends in trace metal concentrations in the decomposing leaves.

The difference in distributions of copper could be related to the higher WRE in EX1 and EX2 than in EX3, and to 4-fold lower copper concentration in epiphytes than did eelgrass leaves (Fig 2C and S3 Table). The higher WRE in EX1 and EX2 would be caused by the high growth of epiphytes at the higher experienced water temperature [18] (see S1 Table).

In the estimation of the WRE, we used four elements, however, the distributions of δ^13^C, N, and δ^15^N were not clear-cut. Because carbon content showed apparent differences between sources compared to variability in each source, it would be the most important measure for estimating accuracy. In addition, because carbon content showed a relatively low standard deviation on an annual average (S3 Table), elements in eelgrass leaves, the use of data from the previous year for EX2 and EX3, would not cause significant problems in estimating accuracy. Similar results between two combinations of elements (Fig 4E), at least, showed the ineffectiveness of nitrogen content and δ^15^N in the estimation of accuracy.

### Effects of leaf decomposition on sediment

Carbon and nitrogen contents increased with increasing δ^13^C in the surface sediment in the eelgrass and reference pools, whereas there was relatively low variability in the bottom sediments. These results demonstrate that the surface sediments originated from sedimentations by marine organisms in both pools, and that the original sediment of the mesocosm experiment still remained in the bottom sediments. The stable isotope ratio of carbon, δ^13^C, in surface sediment in the reference pool had greater variation than in the bottom sediment, and this would be caused by benthic organisms such as marine microphytobenthos, which have between –14 and –17‰ δ^13^C [33]. Conversely, the sedimentation was most likely caused by eelgrass leaves and epiphytes due to the high productivity of eelgrass [28] and comparable biomass in epiphytes (Fig 4) in the eelgrass pool. The mixture of eelgrass leaves, epiphytes, and microphytobenthos would result in δ^13^C in the surface sediment in the pool.

No significant trends with increasing δ^13^C in the surface sediment in the reference pool means that benthic organisms were thought to have similar metal concentrations as the original sediment or no significant biomass. The result in the reference pool can explain that the increase in trace metal concentrations with increasing δ^13^C in the surface sediment in the eelgrass pool would occur due to shed eelgrass leaves with epiphytes. The increase in copper in the eelgrass pool can be explained by higher concentrations in eelgrass leaves than in the original sediment and the increase during leaf decomposition. Lead concentrations were lower in eelgrass leaves than in the original sediment. However, a 10-fold increase in lead during leaf decomposition would increase its concentration in the surface sediment. Zinc and cadmium concentrations in eelgrass leaves decreased during decomposition, and their levels were close to that of the original sediment. However, leaves that still remained that were higher zinc and cadmium would cause the increase with increasing δ^13^C. No significant variations in trace metals among sediments suggest that accumulation of trace metals in the surface sediments may be dependent on the decomposition stages of shed leaves and their biomass.

### Interpretation of the results of mesocosm experiments

The growth of the eelgrass population in this experiment showed similar seasonal patterns of shoot density and shoot size to natural populations in central Honshu, Japan [26], under conditions of sediment and seawater that were taken from Tokyo Bay, where eelgrass is distributed [24, 40]. Therefore, this mesocosm experiment would simulate a natural eelgrass growth and natural eelgrass ecosystem except for physical effects. In natural eelgrass beds where the shed leaves are transported by currents and waves [41, 42], and where the leaves are washed along the coast, which could delay the decomposition of leaves [43], increasing concentrations of trace metals in surface sediment may not occur. However, the seagrass beds, which can efficiently store primary productions [9–11], may accumulate trace metals in the sediment [2]. Although increased copper and lead concentrations in the eelgrass mesocosm were still lower than the concentrations that have been suggested to affect benthic community health [4], they could be achieved that have negative impacts on benthic community health and faunal community structures in the ecosystems [5] if sedimentation by eelgrass production permits the storage of trace metals in sediments.

In summary, mesocosm experiments showed a distribution of trace metal elements and their trends during leaf decomposition. Copper and lead concentrations that increased in the surface sediment of the eelgrass bed would result in their accumulation in decomposed shed eelgrass leaves and epiphytes. Conversely, zinc and cadmium concentrations that decreased during leaf decomposition increased in the surface sediment because of leaves that still contained higher zinc and cadmium. In the natural field conditions under which the primary production of seagrass beds is efficiently stored, eelgrass leaves can be a source of copper and lead cycling in eelgrass ecosystems by accumulating after shedding and decomposing.

## Supporting Information

S1 File Pictures of the mesocosm facilities. (DOCX)

S2 File Detailed descriptions for parameters in the model of leaf decomposition experiment. (DOCX)

S3 File Seawater sampling and analysis for trace metal elements. (DOCX)

S1 Table Summary of decomposition experiments for eelgrass leaves with epiphytes. (DOCX)

S2 Table Number of samples exceeding detection limits for cadmium and lead. (XLSX)

S3 Table Annual element concentrations in normal data in eelgrass leaves, epiphytes, sediments and the Dwass, Steel, Critchlow-Fligner W statistics for the comparison in concentrations between eelgrass leaves, epiphytes, and the surface sediment in eelgrass pool. The concentrations are indicated as mean ± 1 standard deviation. Concentrations in eelgrass leaf were calculated for different annual months. Concentrations in sediment are for surface sediment (SS) and bottom sediment (BS). The subscripts after SS and BS mean eelgrass pool (eel) and reference pool (ref), respectively.
(XLSX)

S4 Table Results of linear model for the element concentrations in log-transformed data and normal data vs. δ^13^C in surface sediment. * *P* < 0.05; ** *P* < 0.01; *** *P* < 0.001.
(XLSX)

S5 Table Summary of predicted concentrations in trace metals at the C content in initial leaves for decomposition experiments (IL) and at the lowest C content in leaves during the experiment (LL). Standard deviation and 95% credible interval are indicated as SD and C.I., respectively. Linear scales are transformed from the predictions in logarithmic scales.
(XLSX)

## References

[1] Lewis MA, Devereux R. Nonnutrient anthropogenic chemicals in seagrass ecosystems: Fate and effects. Environ Toxicol Chem. 2009;28: 644–661. 10.1897/08-201.1 19006414

[2] Serrano O, Davis G, Lavery PS, Duarte CM, Martinez-Cortizas A, Mateo MA, et al Reconstruction of centennial-scale fluxes of chemical elements in the Australian coastal environment using seagrass archives. Sci Total Environ. 2016;541: 883–894. 10.1016/j.scitotenv.2015.09.017 26437357

[3] Macinnis-Ng CMO, Ralph PJ. Variations in sensitivity to copper and zinc among three isolated populations of the seagrass, *Zostera capricorni*. J Exp Mar Biol Ecol. 2004;302: 63–83.

[4] Simpson SL, Batley GB, Chariton AA. Revision of the ANZECC/ARMCANZ sediment quality guidelines. CSIRO Land and Water, 2013, 132 p, https://publications.csiro.au/rpr/pub?pid=legacy:965.

[5] Marín-Guirao L, Atucha AM, Barba JL, López EM, García Fernández AJ. Effects of mining wastes on a seagrass ecosystem: Metal accumulation and bioavailability, seagrass dynamics and associated community structure. Mar Environ Res. 2005;60: 317–337. 15769502 10.1016/j.marenvres.2004.11.002

[6] Schaller J, Brackhage C, Mkandawire M, Dudel EG. Metal/metalloid accumulation/remobilization during aquatic litter decomposition in freshwater: A review. Sci Total Environ. 2011;409: 4891–4898. 10.1016/j.scitotenv.2011.08.006 21907393

[7] Sand-Jensen K. Biomass, net production and growth dynamics an eelgrass (*Zostera marina* L.) population in Vellerup Vig, Denmark. Ophelia. 1975;14: 185–201.

[8] Mukai H, Aioi K, Koike I, Iizumi H, Ohtsu M, Hattori A. Growth and organic production of eelgrass (*Zostera marina* L.) in temperate waters of the Pacific Coast of Japan. I. Growth analysis in Spring-Summer. Aquat Bot. 1979;7: 47–56.

[9] Duarte CM, Marbà N, Gacia E, Fourqurean JW, Beggins J, Barrón C, et al Seagrass community metabolism: Assessing the carbon sink capacity of seagrass meadows. Glob Biogeochem Cycles. 2010;24 10.1029/2010GB003793

[10] Fourqurean JW, Duarte CM, Kennedy H, Marbà N, Holmer M, Mateo MA, et al Seagrass ecosystems as a globally significant carbon stock. Nature Geosci. 2012;5: 505–509.

[11] Watanabe K, Kuwae T. How organic carbon derived from multiple sources contributes to carbon sequestration processes in a shallow coastal system? Glob Change Biol. 2015;21: 2612–2623. 10.1111/gcb.12924 PMC4676932 25880367

[12] Richir J, Luy N, Lepoint G, Rozet E, Alvera Azcarate A, Gobert S. Experimental in situ exposure of the seagrass *Posidonia oceanica* (L.) Delile to 15 trace elements. Aquat Toxicol. 2013;140–141: 157–73. 10.1016/j.aquatox.2013.05.018 23811022

[13] Sanz-Lázaro C, Malea P, Apostolaki ET, Kalantzi I, Marín A, Karakassis I. The role of the seagrass *Posidonia oceanica* in the cycling of trace elements. Biogeosciences. 2012;9: 2497–2507. 10.5194/bg-9-2497-2012

[14] Drifmeyer JE, Thayer GW, Cross FA, Zieman JC. Cycling of Mn, Fe, Cu and Zn by eelgrass, *Zostera marina* L. Am J Bot. 1980;67: 1089–1096.

[15] Lyngby JE, Brix H. Seasonal and environmental variation in cadmium, copper, lead and zinc concentrations in eelgrass (*Zostera marina* L.) in the Limfjord, Denmark. Aquat Bot. 1982;14: 59–74.

[16] Kaldy JE. Carbon, nitrogen, phosphorus and heavy metal budgets: How large is the eelgrass (*Zostera marina* L.) sink in a temperate estuary? Mar Poll Bull. 2006;52: 342–353. 10.1016/j.marpolbul.2005.11.019 16403536

[17] Lyngby JE, Brix H. Heavy metals in eelgrass (*Zostera marina* L.) during growth and decomposition. Hydrobiologia. 1989;176–177:189–196.

[18] Hosokawa S, Nakamura Y, Kuwae T. Increasing temperature induces shorter leaf life span in an aquatic plant. Oikos. 2009;118: 1158–1163.

[19] Harris LA, Buckley B, Nixon SW, Allen BT. Experimental studies of predation by bluefish *Pomatomus saltatrix* in varying densities of seagrass and macroalgae. Mar Ecol Prog Ser. 2004;281: 233–239.

[20] Short FT. Effects of sediment nutrients on seagrasses: literature review and mesocosm experiment. Aquat Bot. 1987;27: 41–57.

[21] Burkholder JM, Mason KM, Glasgow HB. Water-column nitrate enrichment promotes decline of eelgrass *Zostera marina*: evidence from seasonal mesocosm experiments. Mar Ecol Prog Ser. 1992;81:163–178. .

[22] Short FT, Burdick DM, Kaldy JE. Mesocosm experiments quantify the effects of eutrophication on eelgrass, *Zostera marina*. Limnol Oceanogr. 1995;40: 740–749.

[23] Short F, Carruthers T, Dennison W, Waycott M. Global seagrass distribution and diversity: A bioregional model. J Exp Mar Biol Ecol. 2007;350: 3–20.

[24] Hosokawa S, Nakaoka M, Miyoshi E, Kuwae T. Seed dispersal in the seagrass *Zostera marina* is mostly within the parent bed in a protected bay. Mar Ecol Prog Ser. 2015;523: 41–56. 10.3354/meps11146

[25] AIST. Geochemical map of Japan. 2015;9: 6 Available: https://gbank.gsj.jp/geochemmap/zenkoku/periodic_table_riku-umi.htm.

[26] Nakaoka M, Aioi K. Ecology of seagrasses *Zostera* spp. (Zosteraceae) in Japanese waters: a review. Otsuchi Mar Sci. 2001;26: 7–22.

[27] Hibino T, Toyota M, Nishimori D, Hosokawa Y, Tsuruya H. Current field characteristics and ecosystem investigations in Kurihama Bay. Report of the Port and Harbour Research Institute. 1999;38: 29–62, in Japanese with English abstract.

[28] Hosokawa S. A model for measuring leaf growth rate of vegetative shoot in the seagrass, *Zostera marina*. Ecol Model. 2011;222: 105–111.

[29] Cloern JE, Canuel EA, Harris D. Stable carbon and nitrogen isotope composition of aquatic and terrestrial plants of the San Francisco Bay estuarine system. Limnol Oceanogr. 2002;47: 713–729.

[30] Schneider G, Chicken E, Becvarik R. Functions and Datasets to Accompany Hollander, Wolfe, and Chicken—Nonparametric Statistical Methods, Third Edition. 2016. https://cran.r-project.org/web/packages/NSM3/.

[31] R Development Core Team. R: A language and environment for statistical computing. R Foundation for Statistical Computing, Vienna 2014 http://www.r-project.org/.

[32] Takai N, Mishima Y, Yorozu A, Hoshika A. Carbon sources for demersal fish in the western Seto Inland Sea, Japan, examined by δ^13^C and δ^15^N analyses. Limnol Oceanogr. 2002;47: 730–741.

[33] Kuwae T, Beninger PG, Decottignies P, Mathot KJ, Lund DR, Elner RW. Biofilm grazing in a higher vertebrate: The western sandpiper, *Calidris mauri*. Ecology. 2008;89: 599–606. 18459323 10.1890/07-1442.1

[34] Parnell AC, Inger R, Berhop S, Jackson AL. Source partitioning using stable isotopes: Coping with too much variation. PLoS ONE. 2010 10.1371/journal.pone.0009672 PMC2837382 20300637

[35] Clark JS. Why environmental scientists are becoming Bayesians. Ecol Lett. 2005;8: 2–14.

[36] Schaller J, Weiske A, Mkandawire M, Dudel EG. Invertebrates control metals and arsenic sequestration as ecosystem engineers. Chemosphere. 2010;79: 169–173. 10.1016/j.chemosphere.2010.01.015 20132960

[37] Clark JS, Mohan J, Dietze M, Ibanez I. Coexistence: How to identify trophic trade-offs. Ecology. 2003;84: 17–31.

[38] Mantoura RFC, Dickson A, Riley JP. The complexation of metals with humic materials in natural waters. Estuar Coast Mar Sci. 1978;6: 387–408.

[39] Tipping E, Rieuwerts J, Pan G, Ashmore MR, Lofts S, Hill MTR, et al The solid-solution partitioning of heavy metals (Cu, Zn, Cd, Pb) in upland soils of England and Wales. Environ Pollut. 2003;125: 213–225. 12810315 10.1016/s0269-7491(03)00058-7

[40] Tanaka N, Demise T, Ishii M, Shoji Y, Nakaoka M. Genetic structure and gene flow of eelgrass *Zostera marina* populations in Tokyo Bay, Japan: Implications for their restoration. Mar Biol. 2011;158: 871–882.

[41] Abdelrhman MA. Effect of eelgrass *Zostera marina* canopies on flow and transport. Mar Ecol Prog Ser. 2003;248: 67–83.

[42] Nadaoka K, Hino M, Koyano Y. Structure of the turbulent flow field under breaking waves in the surface zone. J Fluid Mech. 1989;204: 359–387.

[43] Nicastro A, Onoda Y, Bishop MJ. Direct and indirect effects of tidal elevation on eelgrass decomposition. Mar Ecol Prog Ser. 2012;456: 53–62.

