# Supplementary material for: Accumulation of Trace Metal Elements (Cu, Zn, Cd, and Pb) in Surface Sediment via Decomposed Seagrass Leaves: A Mesocosm Experiment Using Zostera marina L
Source: PLoS One. 2016 Jun 23;11(6):e0157983. doi: 10.1371/journal.pone.0157983 (PMC4919015; doi:10.1371/journal.pone.0157983)
Supplement: S1 File — (DOCX) [file pone.0157983.s001.docx]

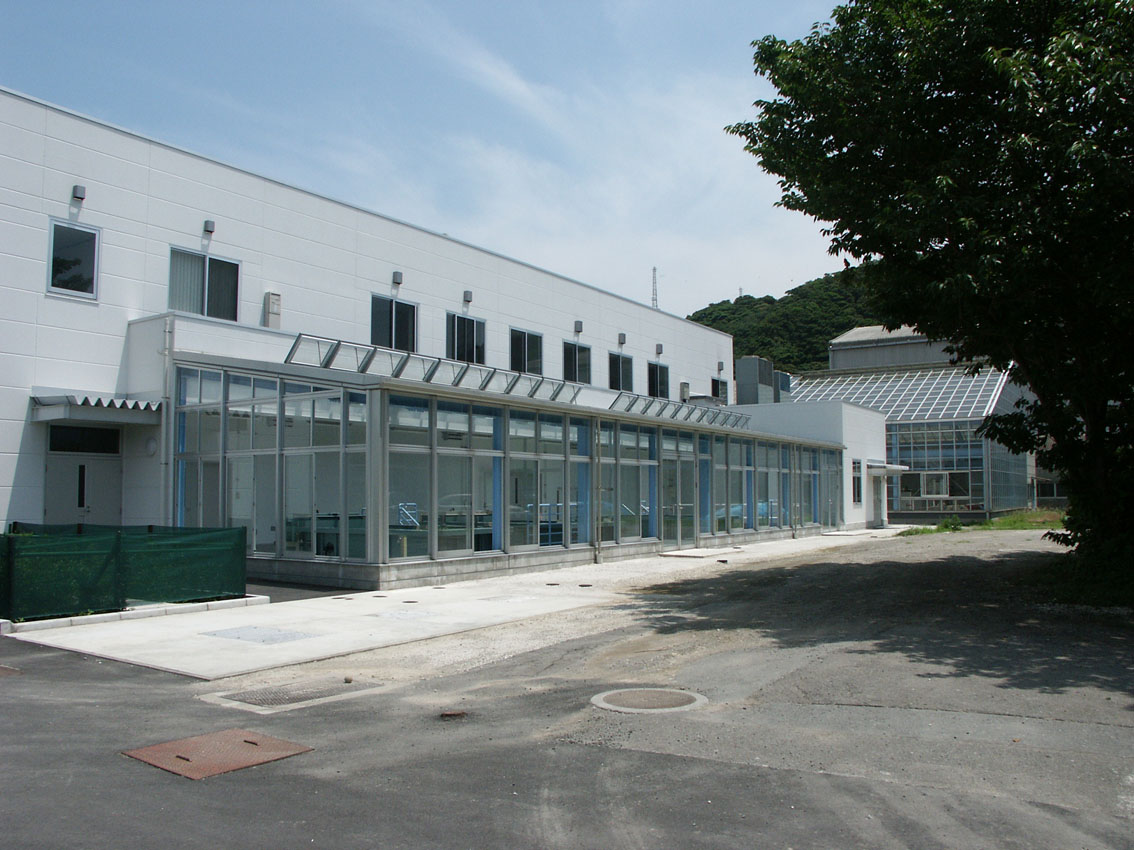


Overview of the mesocosm facilities. Two mesocosm pools are housed in a shed with a glass roof, a glass wall facing south and walls on other sides.


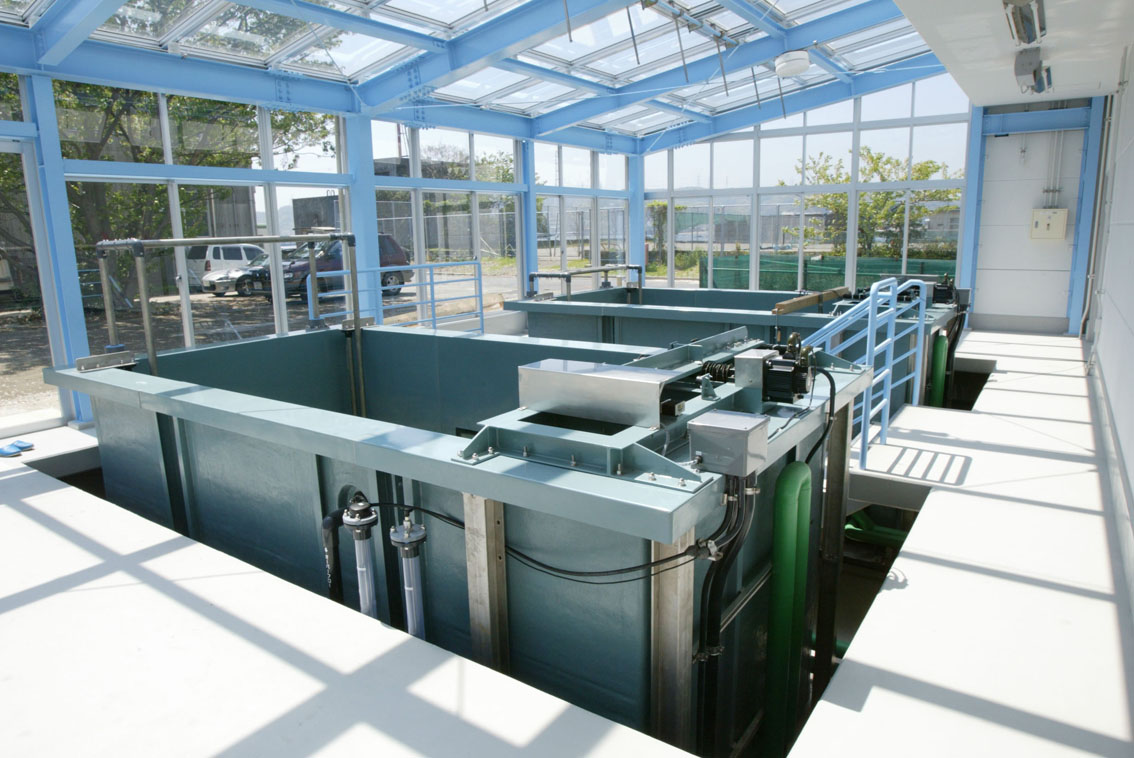


Two mesocosm pools. Eelgrass and reference pools are in the back and front of the picture, respectively.


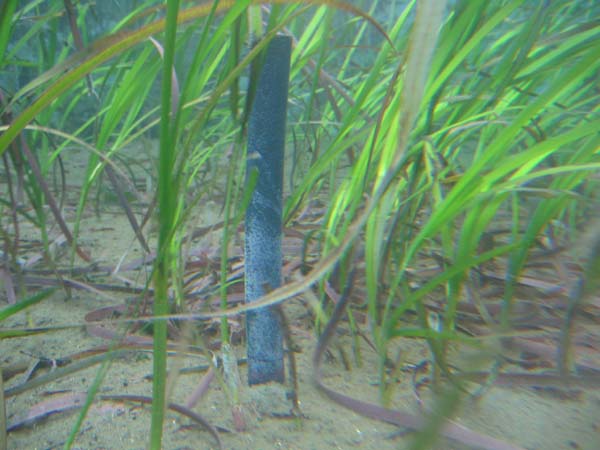

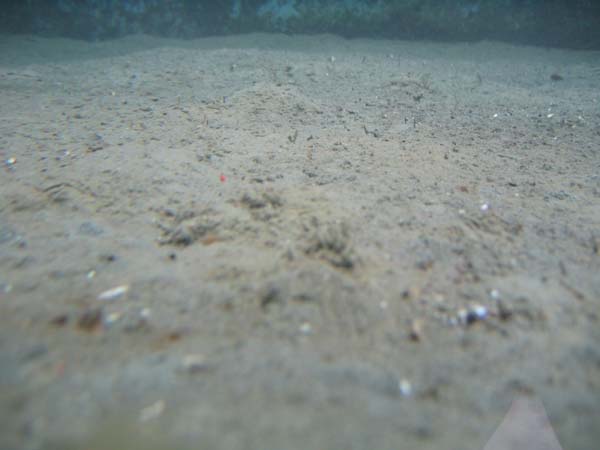


Eelgrass shoots and surface sediment in the eelgrass pool (left) and surface sediment in the reference pool (right). These pictures were taken in February 2005.
