# Supplementary material for: Accumulation of Trace Metal Elements (Cu, Zn, Cd, and Pb) in Surface Sediment via Decomposed Seagrass Leaves: A Mesocosm Experiment Using Zostera marina L
Source: PLoS One. 2016 Jun 23;11(6):e0157983. doi: 10.1371/journal.pone.0157983 (PMC4919015; doi:10.1371/journal.pone.0157983)
Supplement: S2 File — (DOCX) [file pone.0157983.s002.docx]

Parameter ***β****_i_* is assumed to have a multivariate normal distribution (*MVN*), described as:

***β****_i_* ~ *MVN*(***μ***, **Σ**), (S-1)

where ***μ*** is the mean vector (*μ*_1_, *μ*_2_), and **Σ** is the covariance matrix as a follow.

$\Sigma= \left(\begin{matrix} \tau_{1}^{2} & \tau_{12} \\ \tau_{21} & \tau_{2}^{2} \end{matrix}\right)$. (S-2)

The correlation *ρ*_12_ between *β*_1_*_i_* and *β*_2_*_i_* is calculated by *τ*_12_/*τ*_1_/*τ*_2_.

Priors for *σ_i_*^2^ were specified by the Inverse-Gamma distribution (*IG*), *IG*(*a_i_*, *b_i_*), for each experiment *i* of each trace metal element. Prior densities for ***μ*** have *N*(**0**, **D**), and inversed **Σ** have Inverse-Wishart distribution (*IW*), *IW*(**R**, *ν*_0_). Parameters of ***β****_i_*, *σ_i_*^2^, ***μ***, and **Σ** were sampled by the MCMC from posterior distributions as the following equations.

$p(\beta_{i}\left|y_{i}, A_{i}, \right.\sigma_{i}^{2}, \mu, \Sigma) \propto\prod_{j=1}^{nj} N\left(y_{i}\left|A_{i}\cdot\right.\beta_{i}, \sigma_{i}^{2}\right)\times MVN\left(\beta_{i}\left|\mu\right., \Sigma\right)$, (S-3)

$p(\sigma_{i}^{2}\left|y_{i}, A_{i}, \beta_{i}\right., a_{i}, b_{i}) \propto\prod_{j=1}^{nj} N\left(y_{i}\left|A_{i}\cdot\right.\beta_{i}, \sigma_{i}^{2}\right)\times IG\left(\sigma_{i}^{2}| a_{i}, b_{i}\right)$, (S-4)

$p(\mu|\beta_{i}, \Sigma, D) \propto\prod_{i=1}^{ni} MVN\left(\beta_{i}|\mu, \Sigma\right)\times N\left(\mu|0, D\right)$, (S-5)

$p(\Sigma|\beta_{i}, \mu, R, \nu_{0}) \propto\prod_{i=1}^{ni} MVN\left(\beta_{i}|\mu, \Sigma\right)\times IW\left(\Sigma|R, \nu_{0}\right)$. (S-6)

Hyperparameters were set to be relatively non-informative. *a_i_* and *ν*_0_ were 1.0×10^–2^ and 2.0×10^–2^, respectively. They are relatively lower than *n_j_*/2 (= 15) and *n_i_* (= 3), respectively. *b_i_* was 1.0×10^–6^. **D** and **R** were Diag(1.0×10^4^, 1.0×10^4^) and Diag(1.0×10^–4^, 1.0×10^–4^), respectively. Effects of *b_i_*, **D,** and **R** on results were compared with related posterior distributions, which were obtained after calculations; that is, the value that *b_i_* is divided by (*a_i_* + *n_j_*/2), 6.7 × 10^–8^, was compared with posterior *σ_i_*^2^. The value that **D**^–1^ divided by *n_i_*, Diag(3.3 × 10^–5^, 3.3 × 10^–5^), was compared with the diagonal elements of **Σ**^–1^, Diag(1/*τ*_1_^2^, 1/*τ*_2_^2^). **R** divided by (*n_i_* + *ν*_0_), Diag(3.3 × 10^–5^, 3.3 × 10^–5^), was compared with Diag(*τ*_1_^2^, *τ*_2_^2^).
