# Supplementary material for: Accumulation of Trace Metal Elements (Cu, Zn, Cd, and Pb) in Surface Sediment via Decomposed Seagrass Leaves: A Mesocosm Experiment Using Zostera marina L
Source: PLoS One. 2016 Jun 23;11(6):e0157983. doi: 10.1371/journal.pone.0157983 (PMC4919015; doi:10.1371/journal.pone.0157983)
Supplement: S3 File — (DOCX) [file pone.0157983.s003.docx]

Samples of seawater in the eelgrass and reference pools were taken on day of sediment sampling but before sediment sampling to avoid effects of sediment disturbance on the seawater. Seawater was placed in a 1-L bottle and immediately adjusted to a pH between 1 and 2 by adding HNO_3_.

For analysis of Cu, Zn, Cd, and Pb, 100 mL seawater was taken from a sample and digested at 100°C for 10 min with 5 mL nitric acid. The target metals Cu, Zn, Cd, and Pb in the seawater were analysed using inductively coupled plasma mass spectrometry (HP-4500, Agilent Technologies, Santa Clara, CA, USA). The performance of analyses was monitored using a series of internal quality control standards.

Copper, Zn, Cd, and Pb in seawater were detected in both the eelgrass and reference pools (S6 Table).

**S6 Table. Concentrations of Cu, Zn, Cd, and Pb in the water column in eelgrass and reference pools.**

|  | Eelgrass pool | | | |  | Reference pool | | | |
| --- | --- | --- | --- | --- | --- | --- | --- | --- | --- |
|  | Cu | Zn | Cd | Pb |  | Cu | Zn | Cd | Pb |
|  | μg L^−1^ | | | |  | μg L^−1^ | | | |
| Jul 05 | 0.9 | <1 | <0.1 | 0.3 |  | 2.2 | <1 | <0.1 | 0.5 |
| Sep 05 | 1.9 | 4 | 0.3 | 0.4 |  | 1.6 | 1 | 0.1 | 0.2 |
| Dec 05 | 0.3 | 4 | 1.2 | 5.8 |  | 0.9 | 1 | 1.5 | 4.8 |
| Mar 06 | 0.5 | <1 | <0.1 | <0.1 |  | 0.6 | <1 | <0.1 | <0.1 |
