# Supplementary material for: Accumulation of Trace Metal Elements (Cu, Zn, Cd, and Pb) in Surface Sediment via Decomposed Seagrass Leaves: A Mesocosm Experiment Using Zostera marina L
Source: PLoS One. 2016 Jun 23;11(6):e0157983. doi: 10.1371/journal.pone.0157983 (PMC4919015; doi:10.1371/journal.pone.0157983)
Supplement: S1 Table — (DOCX) [file pone.0157983.s004.docx]

|  | Sampling schedule | | | | | | |  | Temperature (°C) | | | | | | |
| --- | --- | --- | --- | --- | --- | --- | --- | --- | --- | --- | --- | --- | --- | --- | --- |
|  | Start day | Days after start day | | | | | |  | Experienced | | |  | Experimental | | |
|  |  | 1st | 2nd | 3rd | 4th | 5th | 6th |  | mean | min | max |  | mean | min | max |
| EX1 | 12th Jul 06 | 0 | 6 | 14 | 26 | 40 | 54 |  | 21.7 | 19.9 | 23.5 |  | 24.9 | 22.0 | 27.1 |
| EX2 | 28th Nov 06 | 0 | 6 | 13 | 26 | 42 | 56 |  | 19.0 | 15.8 | 21.4 |  | 13.3 | 11.3 | 16.0 |
| EX3 | 2nd Apr 07 | 0 | 7 | 14 | 29 | 42 | 56 |  | 13.7 | 11.7 | 16.8 |  | 17.4 | 14.1 | 22.1 |

Experienced temperature is defined as the temperature that eelgrass leaves experienced from the emergence to be the third-youngest leaf.

Experimental temperature is the temperature during decomposition experiments.
